# Synthesis of Polyaniline (PANI) in Nano-Reaction Field of Cellulose Nanofiber (CNF), and Carbonization

**DOI:** 10.3390/polym8020040

**Published:** 2016-02-02

**Authors:** Yuki Kaitsuka, Noriko Hayashi, Tomoko Shimokawa, Eiji Togawa, Hiromasa Goto

**Affiliations:** 1 Division of Materials Science, Faculty of Pure and Applied Sciences, University of Tsukuba, Tsukuba Ibaraki 305-8573, Japan; s-y-kaitsuka@ims.tsukuba.ac.jp; 2 Forestry and Forest Products Research Institute, Tsukuba Ibaraki 305-8687, Japan; tshimo@ffpri.affrc.go.jp (T.S.); togawae@ffpri.affrc.go.jp (E.T.)

**Keywords:** cellulose nano-fiber, ESR, enzyme, polyaniline, solid state NMR, shape preserved carbonization (SPC)

## Abstract

Polymerization of aniline in the presence of cellulose nano-fiber (CNF) is carried out. We used dried CNF, CNF suspension, and CNF treated by enzyme and ultra-sonification to obtain polyaniline (PANI)/CNF as a synthetic polymer/natural nano-polymer composite. The polymerization proceeds on the surface of CNF as a nano-reaction field. Resultant composites show extended effective π-conjugation length because CNF as a reaction field in molecular level produced polymer with expanded coil structure with an aid of orientation effect of CNF. Possibility of PANI β-pleats structure in molecular level of PANI on the CNF is also discussed. SEM observation showed that fine structure is easily obtained by combining PANI with CNF. Carbonization of PANI/CNF allows production of nano-fine form with shape preserved carbonization (SPC).

## 1. Introduction

Cellulose nano-fiber (CNF) has been receiving attention because of interesting mechanical and optical properties [1,2,3,4]. CNF has high adsorption property, and good adhesion between the fibers. Transparent property of CNF was applied for transparent electrodes, display, and bio-functional materials [5,6,7,8]. Super orientation effect of the CNF provides high mechanical strength and high thermal conductivity [9,10,11].

Polyaniline (PANI) is one of the most promising conductive polymers. PANI can be prepared in the water media [12] with no necessity of inert gas. Advantages of simplicity and affinity with water in the synthesis of PANI allow combination with other materials for production of composites [13]. PANI-based synthetic composite has been studied to improve processability and electrical conductivity for applications such as bio systems, sensors, and electronic devices [14,15,16]. Besides, there are some reports about controlling morphology of PANI by preparing composite as template [17,18]. In this study, we carried out synthesis of PANI in the presence of CNF to obtain PANI/CNF as a synthetic polymer/natural polymer composite with nano-structure. Electrically-conductive fibers [19] can be obtained by a simple method, combining PANI with CNF in water media. This paper reports synthesis, chemical structure, surface structure, electron spin resonance (ESR) measurements, electrical conductivity, and carbonization of the PANI/CNF composite.

## 2. Materials and Methods

### 2.1. Materials

Aniline (Wako, Osaka, Japan) and water were distilled prior to use. Sulfuric acid (Nacalaitesque, Kyoto, Japan) and ammonium peroxodisulfate (APS; Kanto Chemical, Tokyo, Japan) were used as received. A 1% cedar-cellulose nanofiber (CNF) suspension was provided by Forestry and Forest Products Research Institute, (Tsukuba, Ibaraki, Japan [20]).

### 2.2. Synthesis

A series of polyaniline/cellulose nanofiber (PANI/CNF) was synthesized with polymerization of aniline as a monomer in the presence of nano-cellulose. PANI is deposited on the surface of the nano-cellulose to form synthetic polymer/natural polymer composite.

PA-NF1-3 were synthesized as follows. Firstly, aniline (monomer), sulfuric acid (55.4 mg, 0.537 mmol) and dried 1% CNF suspension (the quantities are listed in Table 1) were added in distilled water and stirred for 4 h. Then, the mixture was cooled to 0 °C with an ice bath and stirred for 1 h. Ammonium persulfate (APS, 294 mg, 1.29 mmol) dissolved in minimal amount of water was slowly dropped into the mixture containing the monomer and CNF to initiate oxidative polymerization. The polymerization reaction was continued for 16 h. Then, the reaction product was collected by filtration, washed with a large volume of distilled water and a large volume of methanol until the filtrate was colorless. The resultant material was collected by filtration. The cake was dried at 120 °C for 24 h in an oven to obtain a dark green powder as a desired product. The composites thus prepared were abbreviated as PA-NF*n* (PA = polyaniline, NF = nanofiber, *n* = 1–9).

**Table 1:** Preparation of polyaniline/cellulose nanofiber (PANI/CNF) composites.

PANI/CNF	Aniline	Dried 1% CNF ^b^	1% CNF ^b^ suspension	1% CNF ^b^ (EGUS) ^c^	CNF/Aniline	Water	Product
	(mg, mmol) ^a^	(mg)	(mL)	(mg)	(Feed %)	(mL)	(mg)
PA-NF1	99.7, 1.07	150.4	–	–	150.9	10	242.9
PA-NF2	100.0, 1.07	99.8	–	–	99.8	10	194.7
PA-NF3	100.1, 1.07	50.0	–	–	50.0	10	141.9
PA-NF4	206.9, 2.22	–	20.0	–	96.7	–	302.0
PA-NF5	209.3, 2.25	–	10.0	–	47.8	10	239.5
PA-NF6	210.4, 2.26	–	50.	–	23.7	15	249.9
PA-NF7	200, 2.14	–	20	–	100	–	297.3
PA-NF8	200, 2.14	–	20	–	100	–	314.7
PA-NF9	200, 2.14	–	–	20	100	20	312.0

^a^ Sample weight and mole amount; ^b^ Cellulose nanofiber; ^c^ Defibrating cellulose by adding endoglucanase (enzyme) and ultra-sonification.

PA-NF4-6 were synthesized as follows. Aniline and sulfuric acid (110.9 mg, 1.07 mmol) were added into the solution in 1% CNF suspension in the water (the quantities are listed in Table 1). APS (588 mg, 2.57 mmol) was added to the solution and stirred for 16 h at 0 °C. Then, the crude product was collected by filtration, washed with a large volume of distilled water, and a large volume of methanol until the filtrate was colorless. The resultant material was collected by filtration. The cake was dried at 120 °C for 24 h in an oven to obtain a dark green powder as a desired product.

Pure PANI with no CNF suspension was also synthesized under the same conditions of PA-NF1-6, as a reference.

Ultra-sonification was carried out as dispersion treatment for the cellulose nanofibers prior to the polymerization reaction. PA-NF7 was synthesized as follows: aniline as a monomer and sulfuric acid (110.8 mg, 1.07 mmol) were added in 1% CNF suspension in the water (quantities are listed in Table 1) and stirred for 1.5 h. Then, the mixture was cooled to 0 °C with an ice bath and stirred for 1 h. The mixture was ultra-sonificated with a homogenizer, then APS (588 mg, 2.57 mmol) dissolved in minimal amount of water was slowly dropped into the mixture to initiate oxidative polymerization, and ultra-sonification was continued for 2 h. The solution was stirred for 16 h. The crude product was collected by filtration, and washed successively with a large volume of distilled water, and a large volume of methanol. Finally, centrifugation was performed to collect the desired product. Then, the dark green powder was dried at 120 °C for 24 h in an oven. PA-NF8,9 was synthesized as follows: aniline as a monomer and sulfuric acid (110.8 mg, 1.07 mmol) were added in 1% CNF suspension or 1% CNF (EGUS) suspension in the water (quantities are listed in Table 1) and stirred for 1.5 h. Then, the mixture was cooled to 0 °C with an ice bath and stirred for 1 h. The mixture was ultra-sonificated with a homogenizer for 10 min, then APS (588 mg, 2.57 mmol) dissolved in minimal amount of water was slowly dropped into the mixture to initiate oxidative polymerization. After stirring for 16 h, the green powder was obtained with same purification of PA-NF7.

### 2.3. Techniques

Infrared (IR) absorption spectra for the polymers were obtained using a NICOLET iS5 (Thermo Fisher Scientific, Waltham, MA, USA) with the KBr method. Scanning electron microscopy (SEM) observations performed with S-4800 (HITACHI, Tokyo, Japan). X-ray diffraction (XRD) spectra were collected with RINT-2550HF (RIGAKU, Tokyo, Japan). ^13^C solid state NMR spectra were obtained withChemagneticsCMX-300(Varian, Palo Alto, CA, USA). Chemical shifts are recorded in ppm downfield from adamantine (29.5 ppm from tetramethylsilane, SiMe_4_, TMS) as an internal reference. Electron spin resonance (ESR) measurements were recorded on a EMX-T ESR spectrometer (Bruker, Billerica, CA, USA). Electrical conductivity was measured by four-probe method using LORESTA-GP MCP-T610 (Mitsubishi Chemical Analytech, Kanagawa, Japan). Thermogravimetric analysis (TGA) was performed on a EXSTAR7000 (Seiko Instruments, Chiba, Japan). Ultrasonic homogenizer of VP-30s (TAITEC, Saitama, Japan) was used for dispersion of the CNFs.

## 3. Results and Discussion

### 3.1. IR

**Figure 1:**
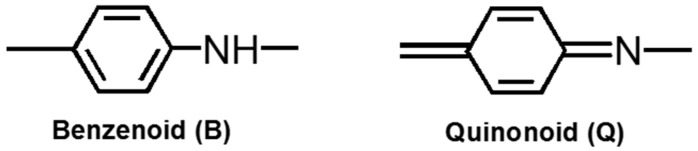

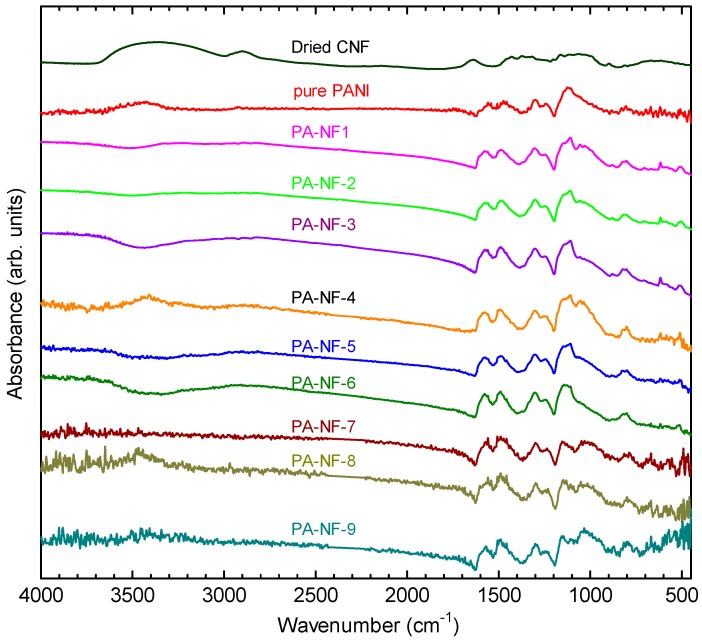
Fourier transform infrared (FTIR) spectra of dried CNF, pure PANI, and PA-NF1, PA-NF2, PA-NF3, PA-NF4, PA-NF5, PA-NF6, PA-NF7, PA-NF8, and PA-NF9.

Infrared (IR) absorption spectra for dried CNF, pure PANI, PA-NF1, PA-NF2, PA-NF3, PA-NF4, PA-NF5, and PA-NF6 are presented in Figure 1. Absorption bands ascribed to the cellulose are observed at 3371 cm^−1^ (broad band, O–H stretching), 2906 cm^−1^ (aliphatic C–H stretching), 1064 cm^−1^ (C–O stretching), and 619 cm^−1^ (C–H out-of-plane). All composites show absorption bonds at 1059 cm^−1^ (C–O stretching). Pure PANI shows characteristic absorptions at 3441 cm^−1^ (N–H stretching), 1554 cm^−1^ (quinonoid (Q) C=C stretching), 1479 cm^−1^ (benzenoid (B) C=C stretching), 1304 cm^−1^ (ν_QBQ_ C–N stretching), 1240 cm^−1^ (ν_BBB_ C–N stretching), and 1124 cm^−1^ (ν_C=N_ Q=N^+^H or B–N^+^H stretching). All composites spectra include characteristic peaks come from PANI at 1574, 1489, 1304, 1249 and 1109 cm^−1^. Furthermore, some absorption peaks at 1574 cm^−1^ (ν_Q_), 1489 cm^−1^ (ν_B_), and 1109 cm^−1^ (ν_C=N_) of the composites are shifted compared to the pure PANI. These shifts can be due to the interaction between PANI and cellulose. The results confirm that the PANI was successfully deposited onto the surface of the nano-cellulose.

### 3.2. Morphology

**Figure 2:**
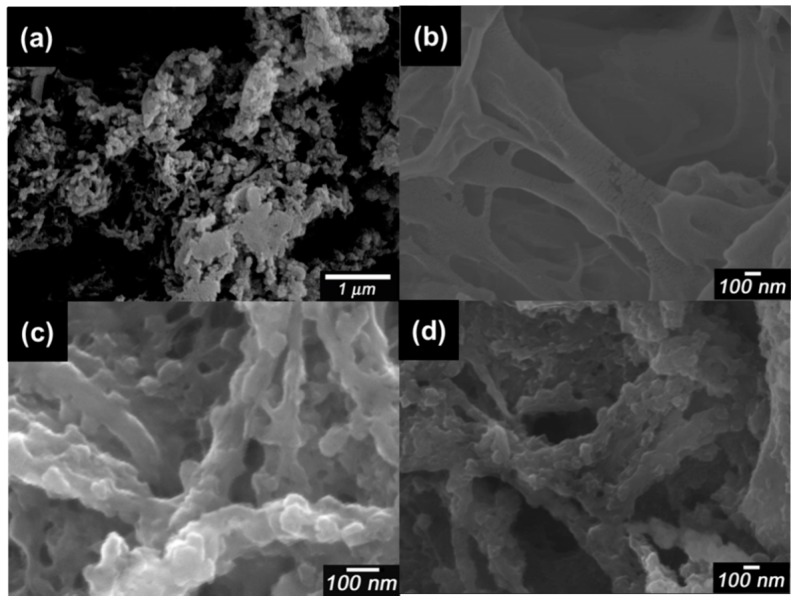
Scanning electron microscopy (SEM) images of pure PANI (**a**); dried CNF (**b**); PA-NF1 (**c**); and PA-NF2 (**d**). PA-NF1 (dried CNF/aniline = 150, feed %). PA-NF2 (dried CNF/aniline = 100, feed %).

**Figure 3:**
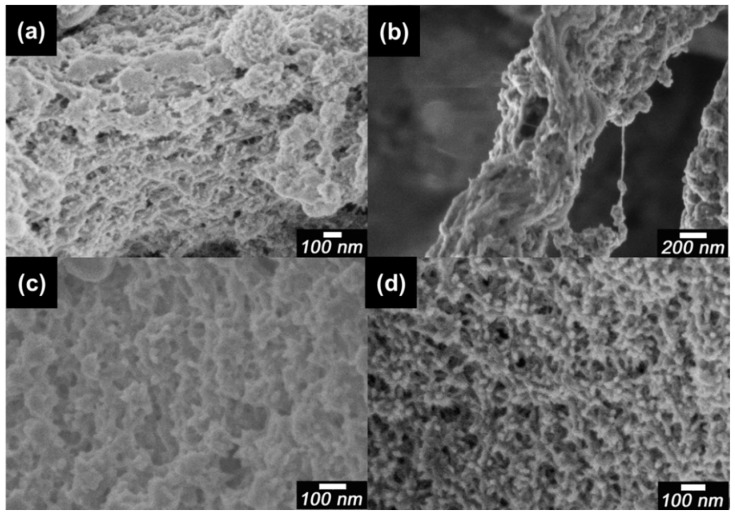
Scanning electron microscopy (SEM) images of PA-NF3 (**a**); PA-NF4 (**b**); PA-NF5 (**c**); and PA-NF6 (**d**). PA-NF3 (dried CNF/aniline = 50, feed %). PA-NF4, PA-NF 5, and PA-NF 6 were prepared by using 1% CNF suspension (CNF/aniline = 100, 50, 25, feed %).

**Figure 4:**
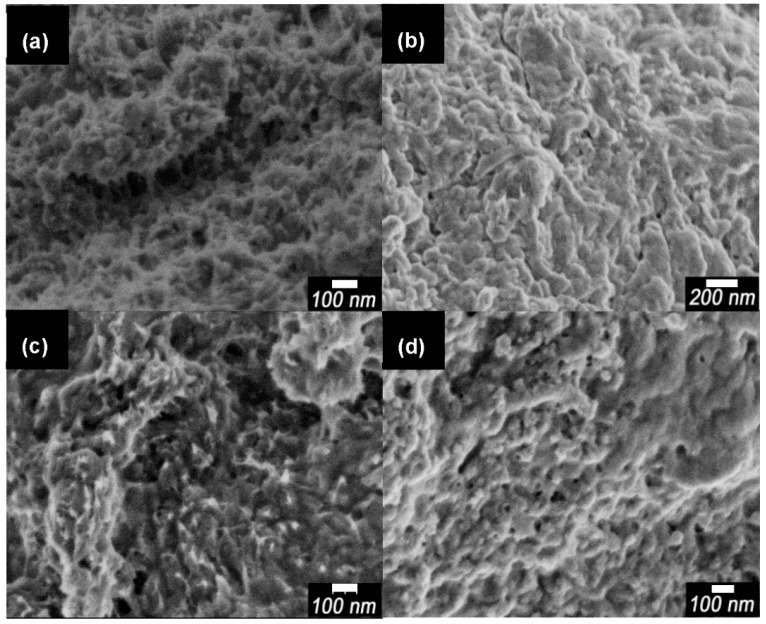
SEM images of PA-NF7 (**a**); PA-NF8 (**b**); PA-NF9 (**c**,**d**). PA-NF7 and PA-NF8 were prepared by using 1% CNF suspension (CNF/aniline = 100, feed %). PA-NF9 was prepared by using 1% CNF (EGUS) suspension (CNF/aniline = 100, feed %).

Scanning electron microscopy (SEM) images of pure PANI, dried CNF, PA-NF1, PA-NF2, PA-NF3, PA-NF4, PA-NF5, and PA-NF6 are shown in Figure 2 and Figure 3. In general, pure PANI shows bulky structure (Figure 2a). Figure 2b showed dried CNF forming aggregates and networks. PA-NF3, PA-NF5, PA-NF6, PA-NF7, PA-NF8, and PA-NF9 show fine structure (Figure 3 and Figure 4). The SEM images indicate that PANI deposits on the cellulose nanofiber.

### 3.3. ESR

**Figure 5:**
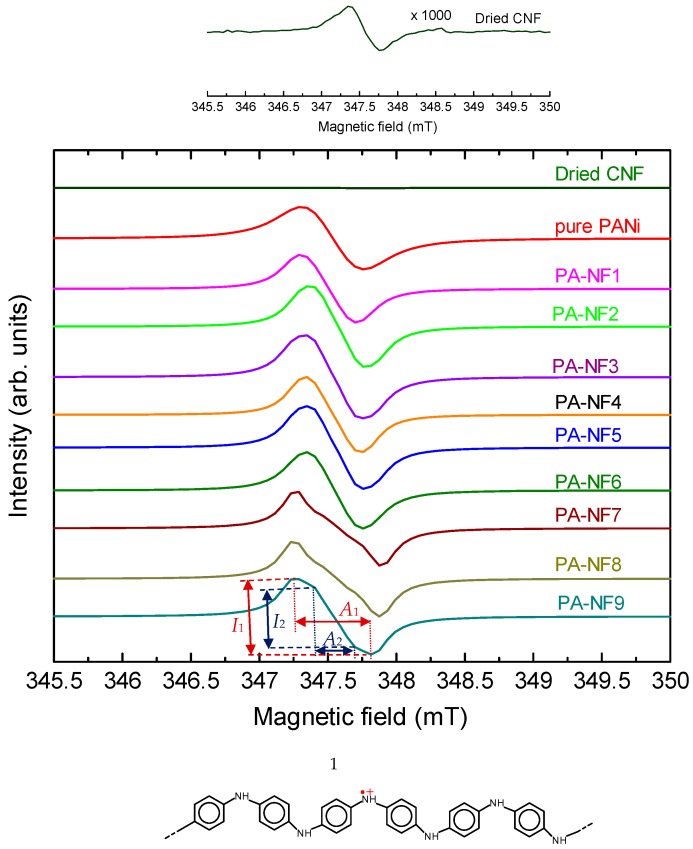
Electron spin resonance (ESR) spectra. (**Top**): Magnification (1000×) of the ESR signal of dried CNF. (**Bottom**): Polarons in PANI.

Electron spin resonance (ESR) spectroscopy measurements for CNF, pure PANI, PA-NF1, PA-NF2, PA-NF3, PA-NF4, PA-NF5, and PA-NF6 were performed (Figure 5). All samples except for CNF displays Lorenz type spectra, indicating the PANI/CNFs have unpaired radicals as charge careers. The radical derived from radical cations distributed along the main-chain is referred to as polarons in the conductive polymers. Therefore, as prepared PANI/CNF is in the doped state. The doping of PANI/CNF with sulfuric acid was carried out during the polymerization process. Table 2 summarizes *g*-value, peak-to-peak line width (Δ*H*_pp_), spin concentration from the ESR measurements, and electrical conductivities of the samples. In general, *g*-value of electron near a nitrogen–hydrogen and carbon–hydrogen bond are to be 2.0054 and 2.0031, respectively. The *g*-values of composites show deviation from the values of free electron (*g* = 2.00023). Narrow Δ*H*_pp_ line width of the PANI/CNF compared with pure PANI indicates delocalization of polarons in the PANI component, suggesting the PANI deposited on the CNF forms a linear shape (expanded coil of PANI) on the molecular level.

**Table 2:** ESR results and electrical conductivity.

PANI/CNF	*g*-Value	Δ*H*_pp_ (mT)	*N*s (Spins/g)	*I*_1_/*I*_2_^a^	σ (S/cm)
pure PANI	2.00435	0.449	1.01×10^22^	–	1.26 × 10^−1^
PA-NF1	2.00375	0.410	5.39×10^19^	–	2.05 × 10^−4^
PA-NF2	2.00355	0.410	6.90×10^19^	–	4.22 × 10^−4^
PA-NF3	2.00354	0.410	7.83×10^19^	–	8.27 × 10^−4^
PA-NF4	2.00357	0.410	5.38×10^19^	–	6.31 × 10^−4^
PA-NF5	2.00346	0.410	7.31×10^19^	–	8.69 × 10^−4^
PA-NF6	2.00351	0.410	7.01×10^19^	–	6.50 × 10^−4^
PA-NF7	2.00412	0.620	7.32×10^21^	2.89	1.39 × 10^−3^
PA-NF8	2.00429	0.620	7.36×10^21^	2.66	6.75 × 10^−3^
PA-NF9	2.00422	0.567	7.83×10^21^	1.22	1.30 × 10^−3^

^a^ *I*_1_/*I*_2_ = (Intensity of the main absorption (*A*_1_))/(intensity of *A*_2_ absorption).*I*_1_, *I*_2_, *A*_1_, and *A*_2_ are defined in Figure 5.

**Figure 6:**
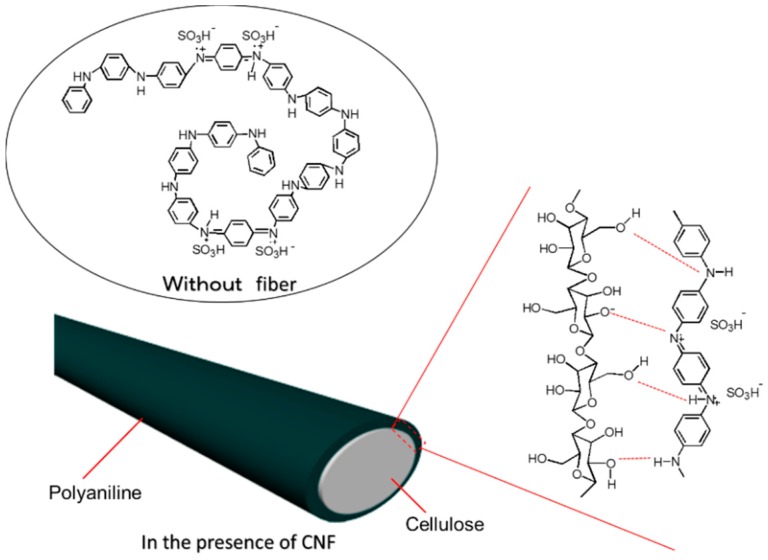
Plausible structure and polymerization mechanism without fiber (**upper**) and on the CNF (**lower**).

The CNF plays a role of linear formed molecular template. Prior to the polymerization reaction, aniline as a monomer is soaked in the CNF with an aid of capillary effect. Polymerization of aniline at the CNF with CNF orientation effect affords linear formed PANI (expanded coil [21]) along the CNF shape (Figure 6). Secondary doping of PANI in *m*-cresol affords structural change from compact coil (entangled coil) to expanded coil form. The conformation change allows development of effective conjugation length. In the present research, expanded coil form of the PANI produced on the CNF, as “textile surface secondary doping”. CNF can be defined as a “nano-reaction field” in this polymerization. In this case, cellulose forms β-pleats structure, therefore, the PANI synthesized in the CNF reaction field can form β-pleats through molecular level transcription. We have studied polymerization in liquid crystals with chemical [22] and electrochemical methods [23]. The resultant polymers obtained with electrochemical polymerization in chiral liquid crystals displays micro-structure, which is quite similar to the matrix liquid crystals. The structure transcription is occurs from molecular level to macroscopic level.

In the present study, structural transcription on a molecular level can occur at the surface of the CNF. Therefore, this result involves molecular structure transcription [24] and molecularly imprinting (MIP) [25]. The secondary doping at nanofiber-surface allows producing PANI with linear structure with improvement of effective π-conjugation length. However, electrical conductivity of the PANI/CNF is low compared with pure PANI because CNF in the composite is insulator. The insulating islands in the PANI/CNF depress the electrical conductivity. PANI with well loosened CNF of PA-NF7, PA-NF8, and PA-NF9 shows higher conductivities than PA-NF1-6 because surface area of the sonificated and enzyme treated samples is increased. Spin concentrations (*N*s) of PA-NF7, PA-NF8, and PA-NF9 are *ca*. 100 times higher than those of the samples with no sonification. *g*-Value of the PA-NF7, PA-NF8, and PA-NF9 is to be *ca*. 2.004. Note that magnification (1000×) of the ESR signal due to the pure CNF after ultra-sonification found existence of unpaired electron signal. This can be due to the fact that the ultra-sonification mechanically produces dangling bond defects in the CNF (mechanoradical).

PA-NF7, PA-NF8, and PA-NF9 display overlapped signals (*A*_1_ and *A*_2_) in the ESR (Figure 5). The *A*_2_ absorption may be related with sonification process or impurities. At the present stage, origin of the *A*_2_ signal in the ESR of PA-NF7, PA-NF8, and PA-NF9 is unknown.

### 3.4. XRD

**Figure 7:**
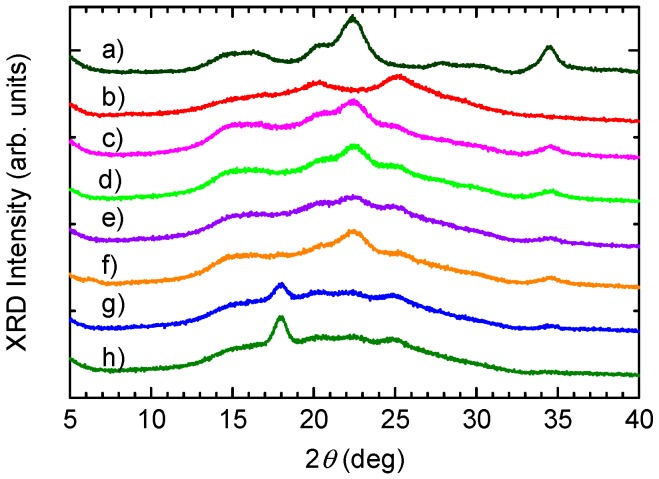
X-ray diffraction profile curves of dried CNF (**a**); pure PANI (**b**); PA-NF1 (**c**); PA-NF2 (**d**); PA-NF3 (**e**); PA-NF4 (**f**); PA-NF5 (**g**); and PA-NF6 (**h**). PA-NF1, 2 and 3 were prepared by using dried CNF (CNF/aniline = 150, 100, 50 (feed %)). PA-NF4, 5, and 6 were prepared by using 1% CNF suspension (CNF/aniline = 100, 50, 25 (feed %)).

X-ray diffraction (XRD) measurements for CNF, pure PANI, PA-NF1, PA-NF2, PA-NF3, PA-NF4, PA-NF5, and PA-NF6 were performed. The results are shown in Figure 7. In the XRD pattern of CNF with cellulose, main peaks located at 22.3°, which can be identified in spectrum for (200) diffraction planes. The broad peak from 14.6° to 16.6° derived from overlapping spectra for (1ī0) and (110) diffraction planes. The spectrum of pure PANI exhibits two main characteristic reflections at 2θ = 20.2° and 25.1°.

These peaks are broad and overlapped with other reflection peaks, suggesting the presence of disordered structures. XRD patterns of the composites also include characteristic peaks come from cellulose and PANI. Furthermore, PA-NF5 and PA-NF6 shows reflections (2θ = 18.0°). This can be due to interactions between PANI and CNF.

**Figure 8:**
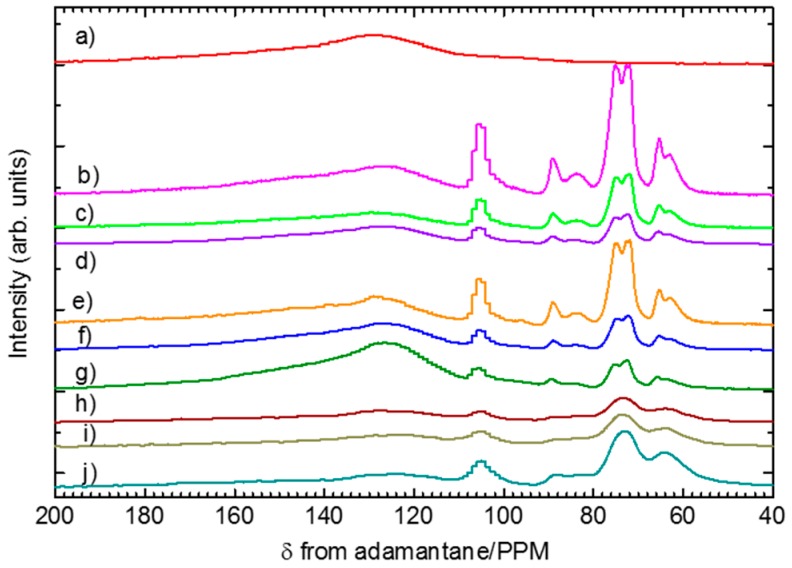
^13^C solid-state NMR spectra of pure PANI (**a**); PA-NF1 (**b**); PA-NF2 (**c**); PA-NF3 (**d**); PA-NF4 (**e**); PA-NF5 (**f**); PA-NF6 (**g**); PA-NF7 (**h**); PA-NF8 (**i**); and PA-NF9 (**j**). PA-NF1, 2, and 3 were prepared by using dried CNF (CNF/aniline = 150, 100, 50 (feed %)). PA-NF4, 5, and 6 were prepared by using 1% CNF suspension (CNF/aniline = 100, 50, 25 (feed %)). PA-NF7 and PA-NF8 were prepared by using 1% CNF suspension (CNF/aniline = 100, feed %). PA-NF9 was prepared by using 1% CNF (EGUS) suspension (CNF/aniline = 100, feed %). Internal standard = adamantane, CH_2_ (δ = 29.5 ppm from tetramethlsilane, TMS).

### 3.5. NMR

^13^C solid state NMR spectra for pure PANI, PA-NF1, PA-NF2, PA-NF3, PA-NF4, PA-NF5, and PA-NF6 were recorded and shown in Figure 8. From these results, pure PANI shows broad peak at around 125 ppm. All composites show peak attributed to both PANI and cellulose from 60 to 110 ppm. The signal intensities due to PANI component are weaker than that of cellulose as increasing amount of CNF in the composite.

### 3.6. Thermal Property

**Figure 9:**
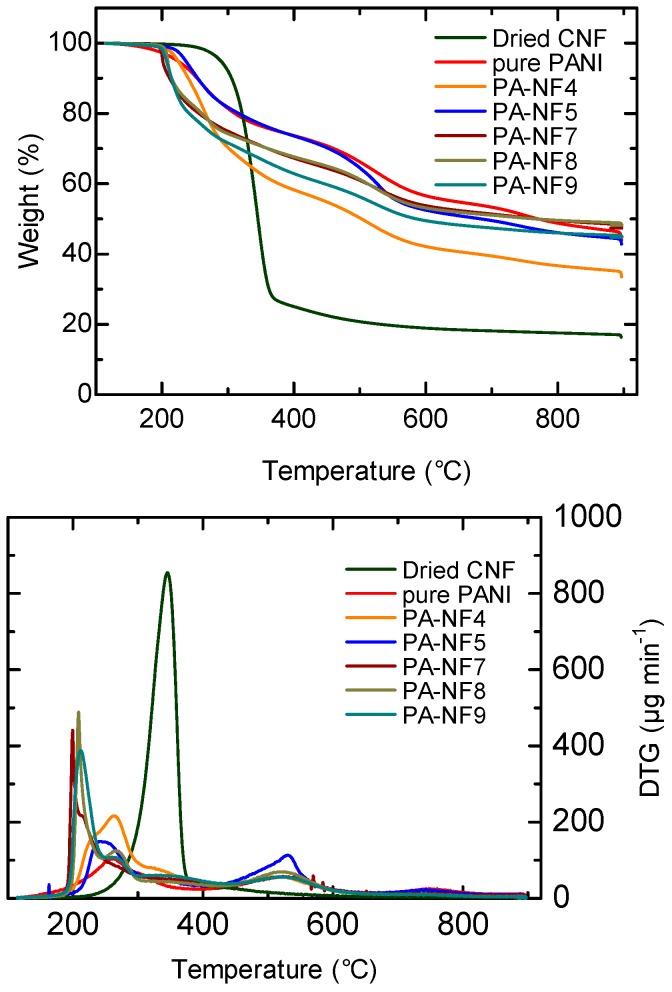
Thermogravimetry analysis (TGA) (**top**) and differential thermogravimetry (DTG) (**bottom**) of dried CNF, pure PANI, PA-NF4, and PA-NF-5, PA-NF7, PA-NF-8, and PA-NF-9.

The thermogravimetric analysis (TGA) and differential thermogravimetry (DTG) for CNF, pure PANI, PA-NF4, and PA-NF5 were carried out to investigate thermal behavior of the samples. All samples were heated at 120 °C to remove residual moisture prior to the measurements. As shown in Figure 9, two main steps of weight loss were observed for pure PANI in the TGA. The first step was observed at 170 °C corresponding to removal of dopant. The second large mass loss derived from the structural decomposition of PANI was occurred in the temperature range of 330–800 °C. The TGA curves of CNF shows two steps of weight loss. The initial weight loss at 250–420 °C is explained by removal of molecular fragments such as O–H and CH_2_–OH groups. The second weight loss is decomposition of cellulose backbone. As for the composites, the weight losses due to decomposition of PANI and CNF are involved. The main degradation element (5% weight reduction temperatures) are summarized in Table 3.

Temperature range of weight reduction of 5% for both composites is slightly higher than that of pure PANI. The TGA curve of PA-NF5 at 250–440 °C is quite similar to that of PANI, suggesting thick PANI layer coated on the CNF surface protects cellulose against thermal degradation.

**Table 3:** TGA results.

Entry	5% Weight reduction temperature (°C)	Residue (%)
CNF	289.0	20.0
pure PANI	223.5	48.0
PA-NF4	224.2	36.6
PA-NF5	235.9	44.8
PA-NF7	201.2	48.4
PA-NF8	208.9	48.8
PA-NF9	206.8	45.7

### 3.7. Shape Preserved Carbonization

**Figure 10:**
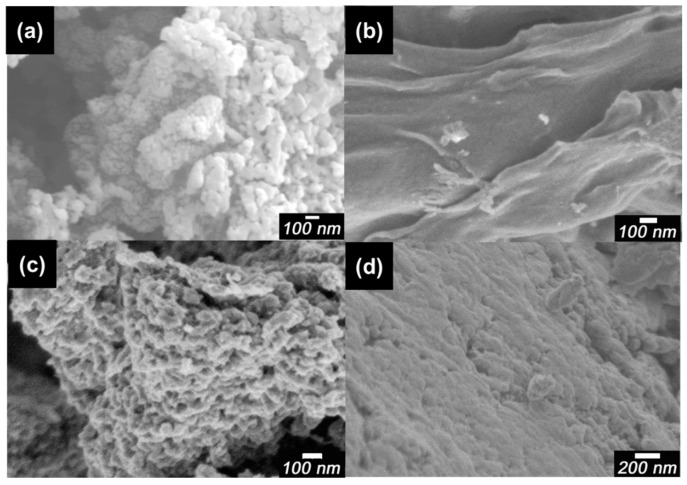
SEM images of carbonized pure PANI (**a**); dried CNF (**b**); PA-NF4 (**c**); and PA-NF5 (**d**). PA-NF4 and PA-NF 5 were prepared by using 1% CNF suspension (CNF/aniline = 100, 50, feed %).

**Figure 11:**
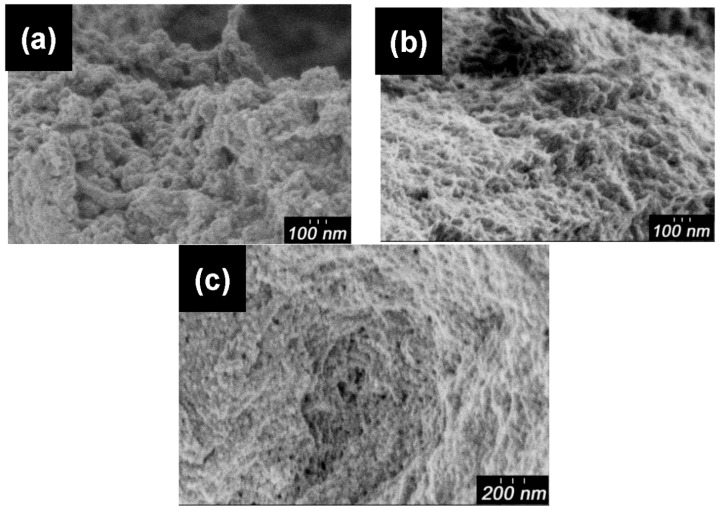
SEM images of carbonized PA-NF7 (**a**); PA-NF8 (**b**); and PA-NF9 (**c**). PA-NF7, and PA-NF8 were prepared by using 1% CNF suspension (CNF/aniline = 100, feed %). PA-NF9 was prepared by using 1% CNF (EGUS) suspension (CNF/aniline = 100, feed %).

Pure PANI, dried (pure) CNF, PA-NF4, and PA-NF5 were carbonized at 1000 °C in an argon atmosphere. The carbons thus prepared shows quite similar structure that of PANI/CNF. In our previous study, we carried out carbonization of PANI with spherical structure to create carbon nano-sphere [12]. In this study, the fiber shape from the dried CNF were preserved after carbonization (Figure 10b). PA-NF4 (Figure 10c) and PA-NF5 (Figure 10d) show granular structure like pure PANI (Figure 10a). The fiber shape of the composites comes from the original form of the cellulose. The shape-preserved-carbonization (SPC) of PANI/CNF allows a production of the fine structure of carbons originating from the polymers at a microscopic level, as shown in Figure 10 and Figure 11. Porous structure of the carbonized samples PA-NF7, PA-NF8, and PA-NF9 is derived from dispersion of CNF through ultra-sonification and use of the enzyme. The fine carbon structure obtained through combination of PANI and cellulose nanofiber can be applied for battery electrodes and hydrogen storage materials having large surface area.

## 4. Conclusions

We prepared polyaniline/cellulose nano-fiber (PANI/CNF) as a synthetic polymer/natural polymer composite with the convenience method. The polymerization reaction proceeds on the surface of CNF with super orientation effect as a reaction field, producing polyaniline having extended effective conjugation due to textile surface secondary doping. CNF as the polymerization reaction field influences conformation and crystallinity of PANI. Then, thermal resistance of composite was improved due to π-stacking of adjacent PANI and interaction between PANI and cellulose. Shape preserved carbonization (SPC) allows production of fine carbons via the original form of PANI/CNF. Note that ultra-sonification produced dangling bond defects in the CNF. Combining with well-defibrated CNF improves the composite’s electrical conductivity. Besides, fiber-shaped and porous structure of composite could be obtained easily.
